# LAFOV PET as an enabling platform for pharmacokinetics-informed digital twins

**DOI:** 10.3389/fmed.2026.1921764

**Published:** 2026-09-15

**Authors:** Bastian Rittmeyer, Matthew Strugari, Sophia Dietrich, Carla González-Avilés, Luis Martí-Bonmatí, Irene Torres Espallardo

**Affiliations:** 1 Department of Biology, Chemistry, and Pharmacy, Freie Universität Berlin, Berlin, Germany; 2 Biomedical Imaging Research Group (GIBI2^30^), Instituto de Investigación Sanitaria La Fe (IIS La Fe), Valencia, Spain; 3 Department of Nuclear Medicine, Hospital Universitario y Politécnico La Fe, Valencia, Spain; 4 Department of Radiology, Hospital Universitario y Politécnico La Fe, Valencia, Spain

**Keywords:** digital twins, LAFOV PET, molecular imaging, pharmacokinetics, physiologically based pharmacokinetic modeling

## Abstract

Digital twins (DTs) have emerged as a promising framework for precision medicine and are a key part of current initiatives to digitalize and innovate the healthcare sector. Medical DTs have shown potential to support more effective and efficient patient care, reduce healthcare costs, and enable novel approaches in drug development. Currently, DTs are primarily informed by structural imaging modalities, genetic information, clinical history, and lifestyle factors. These anatomy-driven twins can capture, for example, tumor burden and morphology in cancer, but lack the pharmacological dimension that ultimately shapes response and toxicity. Long axial field-of-view (LAFOV) PET systems now provide a platform capable of measuring dynamic tracer distribution across all major organs within a single examination, thereby enabling pharmacokinetic (PK) studies that could fill the current gap in DT concepts. The extended axial field of view and the resulting high sensitivity enable comprehensive characterization of patient-specific organ kinetics, potentially advancing DTs for preventive and personalized medicine. In this Perspective article, we discuss how LAFOV PET could complement population-based PK modeling and facilitate the development of PK-informed DTs. We explore its potential implications for selected clinical applications and aspects of drug development. Additionally, we discuss current limitations and outline steps required to translate PK-informed DTs into decision-grade tools for clinical use.

## 1 Introduction

### 1.1 Digital twins

The digital twin (DT) concept was first developed during NASA’s Apollo program where it was used to simulate potential technical failures and support subsequent decision making (1). More broadly, a DT is a virtual representation of a physical entity, such as a machine, factory, or organism. The DT concept is well established in manufacturing and maintenance and was introduced to the healthcare sector in the early 2010s (2). In healthcare, DT concepts encompass operational systems, hospital workflows, and systemic facility management aimed at improving efficiency and resource allocation, whereas in medicine, medical DTs focus more narrowly on biological simulations of patients, organs, or diseases to guide clinical decisions.

A medical DT is minimally characterized as a patient-specific virtual representation of an individual that is dynamically updated with data from its physical counterpart to support clinical prediction and decision making (3, 4). Such data include clinical history, lifestyle factors, genetic information, and structural imaging modalities, such as computed tomography (CT) or magnetic resonance imaging (MRI). Computational models built from these data but lacking iterative updating represent a DT component or precursor rather than a DT itself.

Early DT applications focused on modeling specific organs or conditions such as diabetes (5). These approaches have since evolved to integrate additional and complex data, providing a more complete representation of the individual. DTs have been explored for predicting disease progression and supporting individualized treatment, with the potential to reduce toxicity, enhance efficacy, and improve patient outcomes (6, 7). Additionally, they can help to optimize hospital operations (8) and provide valuable learning platforms, particularly for medical students (9).

The potential of DTs has made them an increasingly prominent concept in the digitalization of medicine and a key component of many innovative strategies for patient treatment and hospital planning. The global market for DT technologies in healthcare is expected to grow with a compound annual growth rate of 25.9% (10), and it is likely that DTs will play a crucial role in current efforts to shift healthcare towards personalized and preventive medicine.

### 1.2 The pharmacokinetic dimension of digital twins

While structural imaging modalities provide undeniably valuable information and genetics offer substantial insights into disease risk, DTs informed by these sources primarily reflect relatively static snapshots of a patient that mainly account for fixed physiological attributes. Together with information from previous hospitalizations and lifestyle data, these inputs have enabled promising DT applications across a range of clinical domains (6–9). However, for truly personalized treatment strategies and reliable predictions of drug uptake, target exposure, metabolism, distribution, and clearance, a detailed understanding of the individual pharmacokinetic (PK) profile is essential.

Patient-specific PK profiles have historically been extremely difficult to measure directly in vivo. Conventional approaches rely heavily on sparse blood sampling, compartmental assumptions, or population-based extrapolations rather than true whole-body measurement (11). Existing models therefore remain mechanistically informed but insufficiently individualized, as they are often derived from prior datasets rather than directly parameterized from patient-specific data. In addition, functional imaging modalities, such as positron emission tomography (PET), have been unable to acquire truly dynamic whole-body data, preventing the simultaneous measurement of multi-organ time–activity curves (TACs) and thereby limiting direct assessment of systemic tracer or drug behavior throughout the body. As a result, the PK component in current DTs is, if implemented at all, commonly inferred rather than measured directly (12).

We propose that individualized PK represents a crucial biological layer for medical DTs, with dynamic long axial field-of-view (LAFOV) PET systems providing the necessary information for next-generation DTs. Although recent work has highlighted the importance of PK-informed and physiology-based DTs, individualized PK information remains insufficiently incorporated into existing DT concepts (4, 12). The consequences of this gap become particularly apparent in oncology, immunotherapy, and radiopharmaceutical therapy, where treatment response and toxicity are strongly determined by how a given agent distributes throughout the body over time (13, 14). The addition of individualized PK information may thus represent the missing functional layer required to unlock the full potential of DTs in medicine.

### 1.3 LAFOV PET enables individualized pharmacokinetic analysis

PET visualizes the in vivo distribution of positron-emitting radiotracers to characterize molecular and physiological processes. Different radionuclide-ligand combinations enable investigation of diverse biological pathways, while quantitative measurements of tracer concentration over time provide the basis for kinetic analysis.

Quantitative dynamic PET predates the introduction of LAFOV PET. Compartmental and graphical kinetic models were established in the 1980s (15, 16), and later work by Meikle et al. demonstrated the direct reconstruction of spatially resolved parametric images from dynamic PET data (17). Population-based and image-derived input functions were likewise developed as alternatives to continuous arterial sampling (18, 19). Furthermore, dynamic whole-body parametric imaging had already been demonstrated on conventional scanners using sequential multi-bed acquisitions (20). LAFOV PET therefore does not introduce kinetic modeling, parametric imaging, or voxel-wise analysis. Its advance lies in combining substantially higher sensitivity with continuous extended axial coverage, enabling simultaneous high-temporal-resolution sampling of blood pools and multiple organs, thereby extending established quantitative methods to the whole-body scale (21).

LAFOV PET systems substantially extend the axial field of view beyond the typical 25–30 cm range of conventional scanners, spanning more than 50 cm in early LAFOV PET concepts to 1–2 m in current systems (22). By convention, LAFOV PET systems typically image from eyes to thighs in a single bed position, whereas total-body PET refers specifically to systems with an axial field of view of 180 cm or greater (23). Despite the lack of standardized terminology, these scanners entered clinical use around 2020 and have since demonstrated benefits across a range of clinical and research applications (24–26). Their high sensitivity and extended axial coverage enable high-temporal-resolution and simultaneous multi-organ imaging suitable for investigating PK processes in vivo (21).

By enabling simultaneous quantitative PK assessment across multiple organs within a single examination, LAFOV PET may, for the first time, provide a practical foundation for generating patient-specific PK profiles suitable for incorporation into medical DTs.

## 2 Linking LAFOV PET tracer kinetics to whole-body pharmacokinetic models

### 2.1 Generation of PET tracer-kinetic parameters and tissue-exposure measures

The ability to acquire dynamic data from anatomically distant organs simultaneously with high sensitivity and ultra-short temporal frames enables quantitative modeling of whole-body physiological pathways. Dynamic LAFOV PET can therefore generate patient-specific whole-body profiles of tracer kinetics and organ- and tissue-level exposure measures.

Dynamic PET data are interpreted using compartmental modeling, which simplifies the body’s complexity by grouping tissues and fluids with similar tracer-kinetic properties into compartments. Standard compartmental models often comprise one to three compartments to represent processes ranging from simple blood-pool distribution to tissue exchange and binding. Tracer concentration is measured over time as a TAC, which is fitted to a compartmental model to estimate the model parameters.

Within a compartmental model, the fitted microparameters are rate constants that describe the underlying tracer kinetics, from which macroparameters are derived (27). For example, in a common two-tissue compartment model, $K_{1}$, $k_{2}$, $k_{3}$, and $k_{4}$ represent tracer delivery to tissue, efflux, trapping or binding, and reverse transport, respectively. From these microparameters, clinically and mechanistically useful metrics can be derived, such as the total distribution volume ($V_{T}$), the net uptake or influx rate ($K_{i}$), or binding potential (BP) in receptor-binding studies (16). Additional tissue-level measures, such as the area under the tissue TAC (${\mathrm{AUC}}_{\mathrm{tissue}}$) (28) and residence times for dosimetry (29), can characterize cumulative tissue exposure and retention, further complementing tracer-kinetic parameters.

The most sophisticated compartmental model is the physiologically based PK (PBPK) model, in which each compartment represents an organ or tissue to mechanistically simulate whole-body drug disposition (30). PBPK models are of increasing importance in precision dosing and drug development (31). Historically, however, PBPK models relied heavily on preclinical data, literature values, and population-based assumptions, as direct parametrization was typically only feasible through organ sampling in animal experiments followed by efforts to translate the results to human models. Consequently, important parameters such as blood flow, tissue partitioning, or clearance were often derived from population averages rather than measured in the individual patient.

The reliance on population averages limits a PBPK model’s value for personalized dosing, response prediction, and drug development. LAFOV PET may help address this limitation by measuring tissue radioactivity concentration over time, thereby providing tracer-specific concentration–time profiles and organ- and tissue-level exposure information rather than PBPK parameters directly. When incorporated into PET–PBPK modeling, these data may constrain estimates of tissue partitioning, permeability, or organ clearance, or serve to validate independent PBPK predictions (32, 33). However, whether a given PBPK parameter can be uniquely identified from PET data depends on the tracer, biological mechanism, and PBPK model structure. Where several parameter combinations can explain the same PET signal, complementary plasma or physiological data and population priors remain necessary for model-informed inference. LAFOV PET should therefore be regarded as a source of patient-specific data for PBPK updating rather than as a direct measurement of PBPK or drug PK parameters. In this way, dynamic LAFOV PET can help calibrate individualized PBPK models to support a more formal representation of patient-specific PK within a DT, as illustrated in Figure 1.

**Figure 1:**
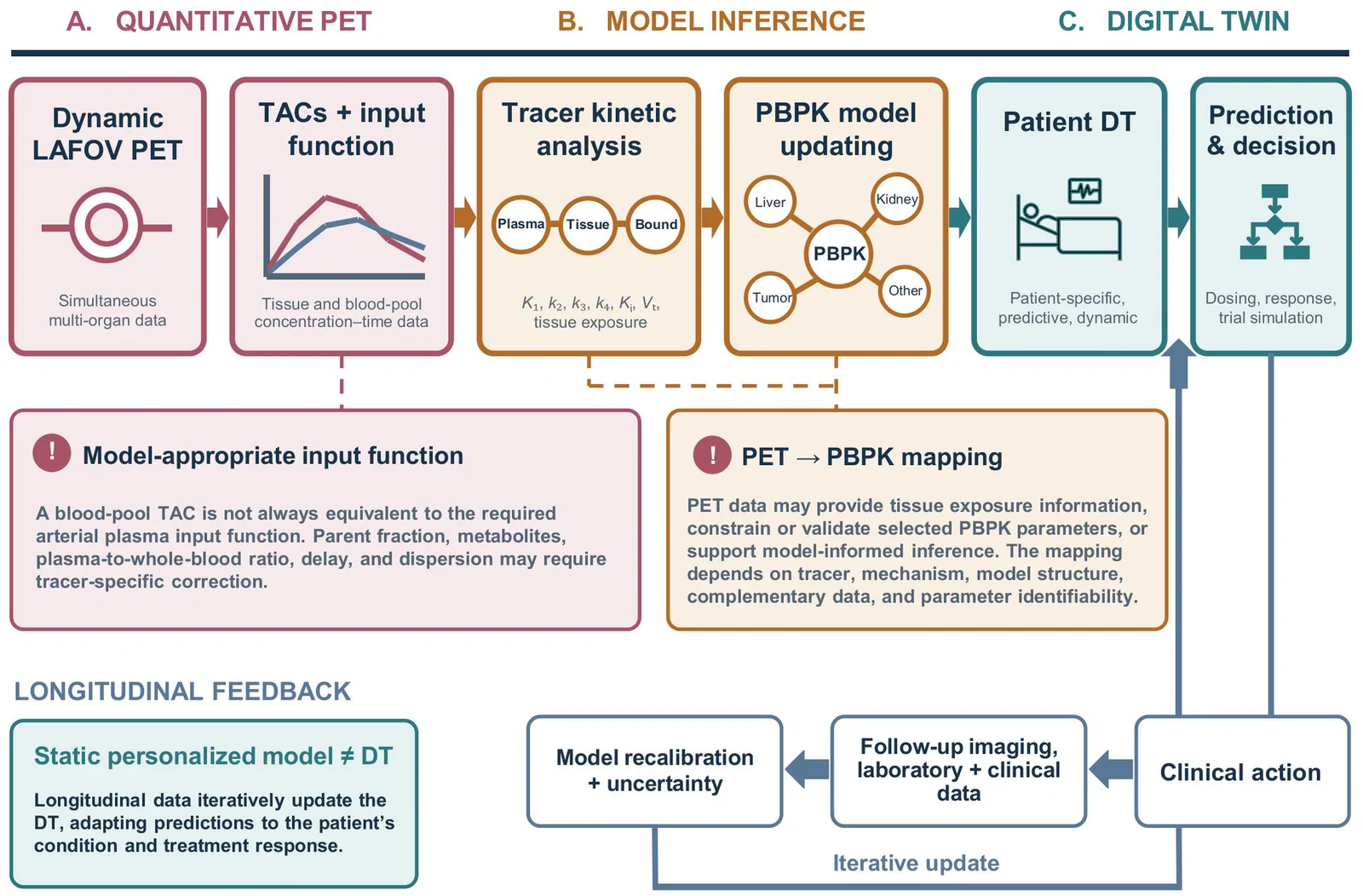
Conceptual framework linking dynamic LAFOV PET, tracer kinetics, PBPK modeling, and medical DTs. (A) Quantitative PET provides patient-specific, time-resolved tracer information that can be processed into TACs, tracer kinetic parameters, and tissue-exposure measures. (B) Model inference uses these data to inform individualized PBPK model updating, subject to limitations related to the input function and PET-to-PBPK mapping. (C) The resulting PK-informed DT supports clinical prediction and decision making. Longitudinal imaging, laboratory, and clinical data enable iterative model recalibration, uncertainty reassessment, and updating.

### 2.2 Requirements for automated PET tracer-kinetic analysis and model integration

Translating the potential of PK-informed DTs into clinically viable workflows with high patient throughput, reproducible results, standardized protocols, and regulatory compliance will require scalable automation of the steps that convert dynamic LAFOV PET data into model-ready, patient-specific tracer-kinetic parameters and tissue-exposure measures.

Reliable organ-level TACs and tracer-kinetic parameters require motion correction, frame-to-frame registration, organ- and target-region segmentation, and a tracer-appropriate input function. An image-derived blood-pool curve is not necessarily equivalent to the required arterial plasma input function. Depending on the tracer and tracer-kinetic model, it may remain necessary to convert whole-blood activity to plasma activity using a tracer-specific plasma-to-whole-blood ratio, obtain limited blood samples, correct for radiometabolites using the parent fraction, or account for delay and dispersion (19, 34, 35). Automated kinetic modeling can then derive regional or voxel-wise tracer-kinetic parameters and tissue-exposure measures from the TACs and input function to inform PBPK or DT models.

Automation of PET-to-parameter workflows is already underway. Dedicated LAFOV PET pipelines automate these steps end-to-end (34), while parallel efforts are accelerating voxel-wise parametric imaging from dynamic PET data, addressing an important computational bottleneck in kinetic analysis (36). Further advances are being driven by artificial intelligence (AI)-based methods for automated multi-organ segmentation and kinetic parameter estimation in dynamic PET (35, 37). In total-body PET, a generative consistency model enabled rapid voxel-wise Bayesian estimation of kinetic parameter distributions and their associated uncertainties from TACs and an arterial input function (37).

To support downstream integration, these workflows should generate outputs with standardized parameter definitions, units, metadata, and uncertainty estimates that can be mapped to PBPK and DT frameworks. Ultimately, the value of LAFOV PET-derived tracer-kinetic and tissue-exposure information for medical DTs will be closely tied to harmonized acquisition and reconstruction protocols, tracer-kinetic and PBPK modeling frameworks, and interoperable output formats.

## 3 Potential impact on healthcare

### 3.1 Diagnosis, treatment planning, and systemic evaluation in oncology

The increased sensitivity of LAFOV PET has been shown to improve image quality and lesion detectability in oncology (38). Beyond these diagnostic benefits, LAFOV PET may support treatment planning, response assessment, and the investigation of systemic cancer effects (39, 40).

Accordingly, LAFOV PET enables a more complete characterization of the initial disease state. In biochemically recurrent prostate cancer, [^68^Ga]Ga-PSMA-11 LAFOV PET/CT showed higher detection rates than short axial field-of-view PET/CT and led to clinically relevant treatment changes, indicating that improved lesion detection is valuable to complement medical DTs (39).

Beyond improved diagnosis, LAFOV PET may also help address the precision dosing gap in oncology. Drug exposure is influenced by organ function, prior medication, comorbidities, tumor burden, and physiological or genetic variability. A review of oncology dose optimization emphasized the importance of integrating PK and pharmacodynamic data with patient-specific factors to support individualized dose and schedule selection (13), while complementary work on tumor drug penetration advocated the inclusion of direct information on tumor exposure (41). However, most clinically applied precision-dosing frameworks remain weakly informed about whole-body tissue exposure because they rely mainly on plasma concentrations, biomarkers, or static imaging, which may not accurately reflect drug exposure at the target site (13, 42).

The lack of patient-specific whole-body tissue exposure information is particularly relevant in the emerging field of theranostics, where patient-specific tumor and normal-tissue exposure is closely linked to treatment efficacy and toxicity. In practice, tumor- and organ-level TACs, tracer-kinetic parameters, and exposure measures derived from dynamic LAFOV PET could constrain patient-specific PBPK model parameters and support simulations of administered activities, dosing intervals, or combination strategies (14).

Quantitative LAFOV PET data may also characterize systemic effects of cancer. A recent study in lung adenocarcinoma and squamous cell carcinoma identified subtype-specific and patient-specific whole-body metabolic connectivity patterns (40). These findings suggest that future models may represent cancer not only as a set of lesions but also as a systemic disease process with distinct molecular alterations. In this framework, PK-informed DTs may also support early response assessment by detecting changes in tracer-kinetic parameters before visible anatomical changes, or aid resistance phenotyping by elucidating lesion-to-lesion heterogeneity, which could help identify biologically distinct sites for targeted biopsy or local treatment. However, these applications remain experimental and require standardized dynamic imaging protocols, repeatable tracer-kinetic parameter estimates, prospective validation against clinical outcomes, and evidence that diagnostic tracer kinetics reflect the behavior of the therapeutic agent (43).

### 3.2 Infectious diseases and immunology

Another potential use case for PK-informed DTs lies in infectious diseases and immunology. LAFOV PET offers particular advantages in these fields, where biological effects are often systemic and require simultaneous multi-organ assessment. Infectious and immune-mediated disorders may involve diffuse processes that are incompletely characterized by anatomical imaging or blood biomarkers alone (44). First-in-human dynamic immunoPET acquired with a LAFOV PET system using [^89^Zr]Zr-Df-crefmirlimab demonstrated quantitative assessment of CD8-positive T-cell distribution and kinetics across lymphoid and non-lymphoid organs in COVID-19 convalescent patients (45). In addition, a prospective proof-of-concept study found that dynamic [^18^F]FDG LAFOV PET provided TACs that differentiated fracture-related infection from sterile inflammation, illustrating how temporal uptake patterns may improve disease phenotyping beyond static uptake measures (46).

In severe infections, treatment response depends on the interplay of pathogen susceptibility, host factors such as renal and hepatic function or immune status, and drug-specific tissue penetration and clearance. A practical PK-informed DT workflow could combine microbiological data and clinical covariates with PET-derived organ kinetics to parameterize PBPK models and simulate whether candidate doses and dosing intervals achieve tissue-specific PK/pharmacodynamic targets (47). Longitudinal imaging, laboratory data, and treatment response could subsequently update the DT during therapy.

Current evidence supports whole-body disease and immune phenotyping (44, 45), but PET-informed antimicrobial dosing remains experimental (47). [^18^F]FDG kinetics reflect glucose metabolism and the host inflammatory response rather than antimicrobial exposure; direct exposure modeling would therefore require imaging of the therapeutic agent or a validated surrogate. Clinical translation further requires pathogen- or drug-specific tracers, standardized dynamic protocols, validation against plasma or tissue measurements, and prospective evidence that model-guided decisions improve clinical outcomes.

### 3.3 Drug development and clinical trial simulation

PK-informed DTs could become valuable tools in drug development, particularly for clinical trial simulation. By linking dynamic LAFOV PET measurements with PBPK models, they could extend conventional plasma-based assessments toward patient-specific simulations of whole-body tissue exposure, target engagement, off-target exposure, clearance, and dosing strategies (26, 42).

One possible workflow could use a radiolabeled drug candidate or validated surrogate in an early-phase cohort to derive organ- and lesion-level TACs and tracer-kinetic parameters that, together with complementary data, could constrain tissue partitioning, binding, and clearance parameters in a PBPK model (26, 42). Virtual cohorts could subsequently be generated by varying relevant characteristics such as organ function, body composition, tumor burden, or target expression. PK-informed DTs could thereby support comparisons of dose levels and intervals, identification of PK phenotypes, earlier go/no-go decisions, and selection of promising trial scenarios for prospective evaluation. Ultimately, this could improve trial design while reducing development time, costs, and patient burden (48).

Realizing this vision will require demonstrating that radiolabeling does not substantially alter drug disposition, determining when tracer or microdose kinetics predict therapeutic-dose behavior, and validating model predictions and their uncertainty prospectively (26). In silico trials should therefore initially complement rather than replace conventional studies. Nevertheless, the broader momentum toward human-relevant methods is reflected by the NIH’s 2026 investment exceeding USD 150 million in laboratory- and computer-based new approach methodologies, while the FDA explicitly discusses in silico trials and patient DTs as emerging tools in clinical research (49, 50).

## 4 Challenges

### 4.1 Technical and computational

The generation of PK-informed DTs from dynamic LAFOV PET data still faces important technical and computational challenges. Quantitative comparability depends on harmonized image reconstruction and greater standardization of PK modeling, while accurate parameter extraction remains limited by patient motion, partial-volume effects, spill-over, and the technical complexity of reliable image-derived input functions. At the same time, dynamic LAFOV PET produces large and heterogeneous datasets that must be integrated with complementary clinical information in a robust and clinically usable way, motivating collaborative efforts such as the Emission Tomography Standardization Initiative to standardize raw data formats in emission tomography (51). The resulting demands on data processing, infrastructure, and personnel may exceed the capacities of many institutions, making automation, scalable software pipelines, and AI-assisted workflows essential for broader translation.

### 4.2 Biological and physiological

In theranostics, the tracer used to assess PK may not perfectly mirror the behavior of the therapeutic compound itself, since even minor molecular differences can alter biodistribution, metabolism, target binding, or clearance. In addition, a patient’s PK profile is not fixed and may change with aging, disease progression, inflammation, organ dysfunction, or medication, meaning that DTs must remain updatable and dynamic. Not all relevant processes can be captured with PET for several reasons. Suitable tracers require identifiable targets, many drugs cannot be easily labeled without changing their properties, and some biological events occur on timescales that are difficult to capture. Lastly, repeated dynamic PET acquisitions for longitudinal PK characterization would require careful consideration of cumulative radiation exposure.

### 4.3 Economic and regulatory

The high cost of LAFOV PET scanners remains a major barrier for hospitals and institutions that plan to acquire such systems. However, recent international health-economic analysis suggests that LAFOV PET may already be cost-effective in standard clinical use (52), implying that its additional use for LAFOV PET-derived PK data and DT applications could further enhance its value by enabling more accurate prediction, more effective treatment strategies, and lower downstream healthcare costs. The performance of the scanners and the quality of the resulting decisions will have to be assessed in multicenter studies that ideally link the DT-derived information to clinical outcomes to satisfy regulatory requirements and qualify for reimbursement. From a regulatory perspective, issues of data handling, storage, interoperability, and data protection must also be addressed to enable secure and compliant studies.

## 5 Conclusion and outlook

Dynamic LAFOV PET provides high-sensitivity, time-resolved measurements of tracer distribution across multiple organs within a single examination. Established kinetic modeling and parametric imaging methods can convert these data into patient-specific TACs, tracer-kinetic parameters, and tissue-exposure measures that can inform PBPK models. These capabilities establish LAFOV PET as an enabling technology for integrating individualized PK information into medical digital twins.

The next step is to translate this technical capability into clinically actionable PK-informed DTs. What remains experimental is not the acquisition of multi-organ kinetic data itself, but its integration into iteratively updated models that quantify uncertainty, predict therapeutic tissue exposure, and prospectively inform dosing or treatment selection. Applications in oncology, infectious diseases, theranostics, and drug development provide promising starting points, although current evidence is largely based on feasibility studies and limited patient cohorts.

Clinical translation will require establishing which PET-derived tracer-kinetic parameters are sufficiently repeatable and clinically actionable, under which conditions tracer kinetics can represent drug or radiopharmaceutical behavior, and how dynamic acquisitions, input functions, reconstruction methods, and kinetic models should be harmonized across centers. Further research must also establish how imaging data should update PBPK models, how predictive uncertainty should be communicated, and whether model-informed decisions improve patient outcomes in prospective multicenter studies.

Altogether, the ability of LAFOV PET to characterize simultaneous multi-organ kinetics provides the technological foundation for linking measured multi-organ tracer kinetics and tissue exposure with predictive computational models. With continued standardization, validation, and integration, LAFOV PET-enabled PK-informed DTs could advance from proof-of-concept frameworks into decision-grade tools for personalized medicine.
